# Clinical differences between *Raoultella* spp. and *Klebsiella oxytoca*

**DOI:** 10.3389/fcimb.2024.1260212

**Published:** 2024-06-03

**Authors:** Sofia K. Mettler, Nipith Charoenngam, Robert C. Colgrove

**Affiliations:** ^1^ Faculty of Medicine, University of Zurich, Zurich, Switzerland; ^2^ Department of Medicine, Mount Auburn Hospital, Cambridge, MA, United States; ^3^ Harvard Medical School, Boston, MA, United States; ^4^ Mahidol University, Faculty of Medicine Siriraj Hospital, Bangkok, Thailand

**Keywords:** *Raoultella*, *Klebsiella oxytoca*, antibiotic, resistance, susceptability, bacteremia, intensive care

## Abstract

**Purpose:**

*Raoultella* spp. is a genus of bacteria that is known to be closely related to *Klebsiella*. It has been debated whether *Raoultella* should be reclassified as a subgroup of *Klebsiella*. The aim of this study is to compare clinical aspects of *Raoultella* and *Klebsiella oxytoca*, a species of *Klebsiella* that is known to be bacteriologically similar to *Raoultella* spp.

**Methods:**

Using data collected at a tertiary care hospital in the United States, we identified 43 patients with *Raoultella* infection and 1173 patients with *Klebsiella oxytoca* infection. We compared patient demographics (age and sex), hospitalization status, isolation sites and antibiotic resistance profiles between the two species.

**Results:**

There was no significant difference in patient demographics between the two bacteria species. The proportions of intensive care unit (ICU) admission were higher among patients with *Raoultella* infection (p=0.008). The most common site of isolation was urine for both species (39.5% of all patients with *Raoultella* spp. vs. 59.3% for *K. oxytoca*). The second most common site of isolation was blood stream for *Raoultella* spp. (23.3%) and respiratory tract for *K. oxytoca* (10.8%). Except for the high proportion of resistant isolates of *Raoultella* spp. for Trimethoprim/sulfamethoxazole, the antibiotic susceptibility profiles were similar between the two bacteria species. Both were susceptible to ciprofloxacin and meropenem.

**Conclusion:**

While there are no significant differences in the patient demographics and antibiotic susceptibility profiles between *Raoultella* spp. and *K. oxytoca*, *Raoultella* may cause more serious infection requiring ICU admissions. Also, *Raoultella* may cause blood stream infection more frequently than *K. oxytoca*.

## 1 Introduction

*Raoultella* spp. are Gram-negative encapsulated aerobic rods that belong to the family Enterobacteriaceae. This organism was initially classified in cluster II of genus *Klebsiella* as *Klebsiella ornithinolytica* until 2001, when cluster II of the genus Klebsiella was renamed to its own genus, *Raoultella*, named after Didier Raoult, a French physician/microbiologist (Hajjar et al., 2020).

*R. ornithinolytica* and *R. planticola* can be found naturally in water environments, fish and insects and are known to cause uncommon infections in humans (Hajjar et al., 2020; Pi et al., 2020; Chen et al., 2021). There are a few recent reports of *Raoultella* colonization and infection in children and adults of varying health status (Naganathan and Amin, 2018; Hajjar et al., 2020; Pi et al., 2020; Chen et al., 2021). Given the rarity of its infection, data on the antimicrobial resistance profile of this organism is relatively limited. It was reported in four university hospitals in France that a significant proportion of *R. ornithinolytica* isolates were resistant to ceftriaxone (4%), quinolones (6%) and trimethoprim/sulfamethoxazole (13%) (Seng et al., 2016).

There have been debates as to whether *Raoultella* spp. should be taxonomically reclassified as a subgroup of the genus *Klebsiella*. A recent genetic analysis of *Raoultella* and *Klebsiella* suggested that they are genetically close enough to belong to one genus (Ma et al., 2021). *Klebsiella oxytoca*, an enteric flora with emerging pathogenic potential, is so similar to *Raoultella* in its morphology and growth pattern that *R. ornithinolytica* can be sometimes misclassified as *Klebsiella oxytoca* (Alves et al., 2006; Park et al., 2011). It remains unclear whether *Raoultella* spp. should be treated differently from *Klebsiella* in clinical settings.

The objective of this study is to compare clinical aspects of *Raoultella* spp. and *Klebsiella oxytoca*. In addition, we analyzed the antibiotic resistance profile of *Raoultella* spp. and compared it to that of *Klebsiella oxytoca* to help guide the antimicrobial management of this uncommon organism.

## 2 Materials and method

### 2.1 Data source

Our study is based on data collected at an academic tertiary care center in Boston, Massachusetts, between 2008 and 2019 (Johnson et al.). This database, MIMIC-IV, contains de-identified clinical data of patients who were seen at the Emergency Department (ED) or admitted to one of the Intensive Care Units (ICU) during the study period. Patients who were hospitalized after presenting to the ED were included in the data, but those who were hospitalized through other routes bypassing the ED (such as hospital-to-hospital transfers) were not included, unless they were admitted to the ICU. As our study is a retrospective analysis of publicly available data, an individual ethics committee approval was waived.

### 2.2 Patients and inclusion criteria

**Figure 1:**
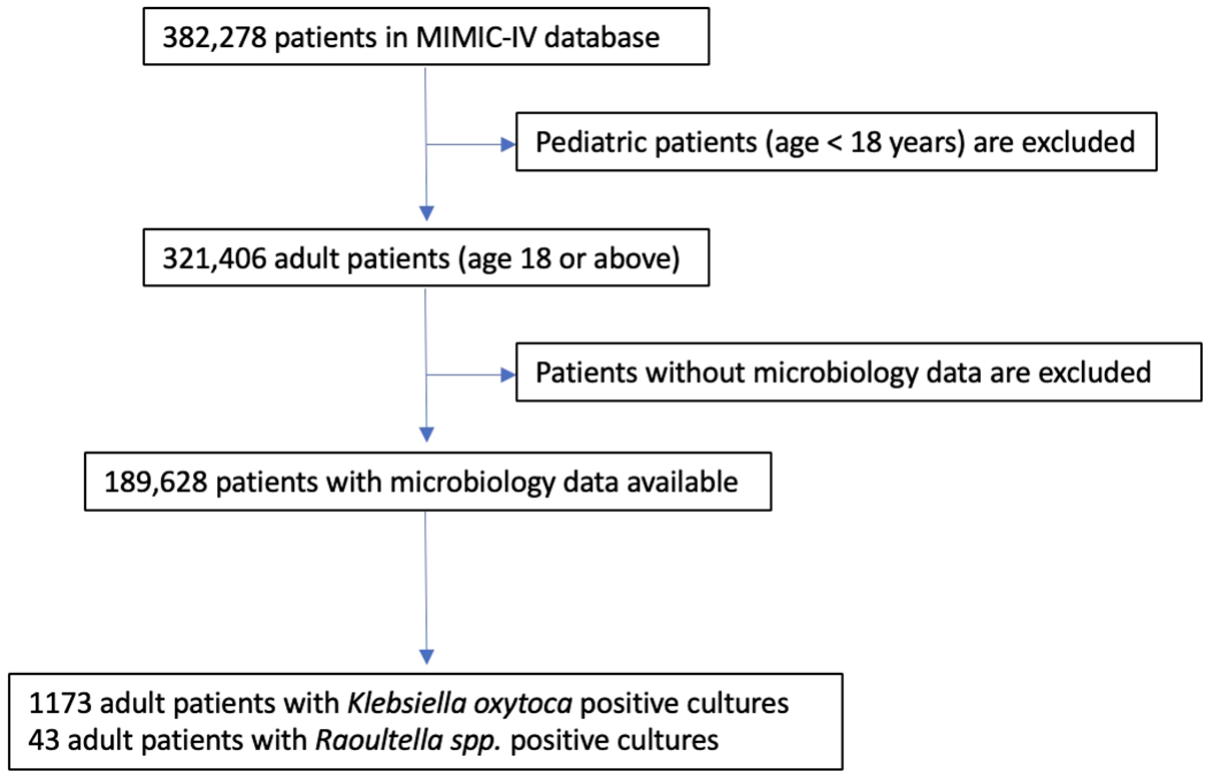
Inclusion flow chart.

The patient list of the utilized database contains 382,278 unique individuals. Pediatric patients are excluded, and only adult patients (18 years-old or older) are included in our study. A flow chart of the inclusion criteria is shown in **Figure 1**.

### 2.3 Microbiology data

The database contains all microbiology laboratory events of the above patient population recorded in the hospital electronic health record systems. The microbiology specimens were mostly collected at the ED, during a hospitalization or during an ICU stay. In rare cases, some specimens may have been collected at an affiliated outpatient office and the results are shared in the system when patients visited ED or are admitted to ICU. The data include specimen collection time, specimen source, types of laboratory tests, organisms detected, antibiotic susceptibility studies and MIC dilution values.

We analyzed all microbiology events where *Raoultella* spp. or *Klebsiella oxytoca* were detected. The *Raoultella* species detected were either *R. ornithinolytica* or *R. planticola*.

### 2.4 MIC dilution values and antibiotic susceptibility interpretation

We re-interpreted the susceptibility studies of each antibiotic agent, using the most recent guidelines and MIC breakpoints published by the Clinical Laboratory Standards Institute (Lewis, 2022). When the MIC dilution value is missing while the original interpretation of susceptibility was resistant, we considered the new interpretation to be also resistant.

The proportions of missing values by each antibiotic agent and its original susceptibility interpretation are available in the **Supplementary Material**.

### 2.5 Statistical methods

For descriptive statistics, numbers of unique specimens and numbers of unique patients are reported separately. Descriptive statistics of patients’ age are computed at the specimen level. For example, if a patient had two specimens collected, one at age 30 and another at age 50, both ages were counted at the average age. Sex statistics are computed at the patient level. P-values are computed using two sample t-tests for continuous variables and two-sample z-tests for proportions. Two sample t-tests or two sample z-tests are performed only on the patient level but not on the specimen level.

The overall antibiotic susceptibility is defined as the number of sensitive specimens to the given antibiotic divided by the number specimens tested for the antibiotic. Each antibiotic susceptibility interpretation at the specimen level, not at the patient level, is used to compute the overall antibiotic susceptibility. In order to avoid bias caused by repeated bacterial cultures within the same patient, the 95% confidence interval for the overall antibiotic susceptibility is computed using a cluster bootstrap method. Each patient is considered to be a cluster. Five thousand bootstrap samples are drawn for each antibiotic and its susceptibility is computed for each bootstrap sample. The 95% bootstrap confidence interval is given by the 2.5^th^ and 97.5^th^ percentiles of the bootstrap estimates.

All statistical analyses are performed using the statistics software R.

## 3 Results

### 3.1 Patient demographics

**Table 1** summarizes the numbers of patients and specimens, patient demographics (age and sex) and hospitalization status. A total of 51 specimens obtained from 43 patients grew *Raoultella* spp. and 1613 specimens from 1173 patients grew *Klebsiella oxytoca*. Among adult patients (18 years old or older), the average age at the time of positive culture was 67 years for *Raoultella* spp. and 67.5 years for *K. oxytoca* (p-value = 0.8). The proportion of female patients was higher among those with *Raoultella* positive cultures (24 patients, 55.8%) than among those with *K. oxytoca* positive cultures (572 patients, 48.8%). However, this difference was not statistically significant (p-value = 0.5).

**Table 1:** Summary of data and patient demographics.

	*Raoultella* spp.	*Klebsiella oxytoca*	p-value
Number of patients	43	1173	–
Number of specimen	51	1613	–
Average age (adults 18+)	67	67.5	0.8
Age quantiles (adults 18+)	25%: 57.5 50%: 67 75%: 77.5	25%: 57 50%: 69 75%: 79	–
Number of female patients	24 (55.8%)	572 (48.8%)	0.5
Number of patients whose specimen is collected **during or within 48 hours** prior to hospitalization	37 (86.0%)	879 (74.9%)	0.1
Number of patients whose specimen is collected **during or within 48 hours** prior to ICU admission	19 (44.2%)	291 (24.8%)	0.007
Number of specimens collected **during or within 48 hours** prior to hospitalization	45	1202	–
Number of specimens collected **during or within 48 hours** prior to ICU admission	24	434	–

Two group means and proportions are compared using two sample t-tests and two sample z-tests, respectively.

Among 43 patients with *Raoultella* positive cultures, 37 patients (86.0%) were hospitalized or became hospitalized within 48 hours of specimen collection. This proportion of hospitalization was lower among patients with *K. oxytoca* positive cultures (879 patients, 74.9%), but the difference did not reach statistical significance (p-value = 0.1). For ICU admissions, however, patients with *Raoultella* positive cultures were significantly more likely to be admitted to ICU at the time of or within 48 hours of specimen collection compared to patients with *K. oxytoca* positive cultures (44.2% vs. 24.8%, p-value = 0.007).

Results only including blood stream infection are available in the **Supplementary Material**.

### 3.2 Culture sites

The numbers and proportions of culture sites are shown in **Table 2A**. For *Raoultella* spp., the most common culture site was urine (30.9%), closely followed by blood (29.1%) and respiratory tract (21.8%). *K. oxytoca* was predominantly found in urine (52.6%), followed by respiratory tract (15.8%) and blood (12.4%). The proportion of urinary tract isolation was significantly higher among patients with *K. oxytoca* than among patients with *Raoultella* (p-value = 0.01). The proportion of blood stream infection was significantly higher among *Raoultella* patients than among *K. oxytoca* patients (p-value = 0.02). Other rarer sites included wound, bile, and peritoneal fluid.

**Table 2A:** Culture sites.

Culture site	*Raoultella* spp.		*Klebsiella oxytoca*		P-values
	Number of specimens	Number of patients	Number of specimens	Number of patients	
Urine	17 (33.3%)	17 (39.5%)	859 (53.3%)	696 (59.3%)	0.02
Blood	16 (31.4%)	10 (23.3%)	203 (12.6%)	127 (10.8%)	0.02
Respiratory	8 (15.7%)	7 (16.3%)	240 (14.9%)	153 (13%)	0.7
Wound	4 (7.8%)	3 (7%)	116 (7.2%)	105 (9%)	0.9
Bile	2 (3.9%)	2 (4.7%)	34 (2.1%)	28 (2.4%)	0.7
Peritoneal fluid	2 (3.9%)	2 (4.7%)	15 (0.9%)	11 (0.9%)	0.1
Joint fluid	–	–	4 (0.2%)	2 (0.2%)	–
Pleural fluid	–	–	4 (0.2%)	4 (0.3%)	–
CSF	–	–	1 (0.1%)	1 (0.1%)	–
Other	2 (3.9%)	2 (4.7%)	137 (8.5%)	113 (9.6%)	–

P-values are computed comparing the proportions of patients in each group between Raoultella spp. and Klebsiella oxytoca using two-sample z-tests.

We compared the stratified results by the clinical context (ICU admission, hospitalization and no hospitalization). **Table 2B** shows comparison of cultures sites among patients who were admitted to ICU at the time of or within 48 hours of specimen collection. Comparison tables of culture sites among hospitalized patients and those who were not hospitalized are available in the **Supplementary Material**. When only severely ill patients who needed to be admitted to ICU are compared, blood stream infection accounted for the majority (6 patients, 31.6%) among *Raoultella* patients, while respiratory infection was most common (111 patients, 38.1%) among *K. oxytoca* patients. Among *K. oxytoca* patients admitted to ICU, the proportion of blood stream infection was lower with 16.2% than 31.6% among *Raoultella* patients. However, this difference was not statistically significant (p-value = 0.2).

**Table 2B:** Culture sites stratified by the context of specimen collection.

Culture site	*Raoultella* spp.		*Klebsiella oxytoca*		P-values
	Number of specimens	Number of patients	Number of specimens	Number of patients	
Blood	10 (41.7%)	6 (31.6%)	75 (17.3%)	47 (16.2%)	0.2
Respiratory	6 (25%)	5 (26.3%)	183 (42.2%)	111 (38.1%)	0.4
Urine	5 (20.8%)	5 (26.3%)	110 (25.3%)	101 (34.7%)	0.6
Bile	2 (8.3%)	2 (10.5%)	13 (3%)	12 (4.1%)	0.5
Peritoneal fluid	1 (4.2%)	1 (5.3%)	2 (0.5%)	2 (0.7%)	0.4
Wound	–	–	13 (3%)	13 (4.5%)	–
Pleural fluid	–	–	3 (0.7%)	3 (1%)	–
Joint fluid	–	–	2 (0.5%)	1 (0.3%)	–
CSF	–	–	1 (0.2%)	1 (0.3%)	–
Other	–	–	32 (7.4%)	29 (10%)	–

Among patients who are admitted to ICU within 48 hours of specimen collection, or whose specimens are collected during an ICU admission.

### 3.3 Antibiotic resistance

**Figure 2:**
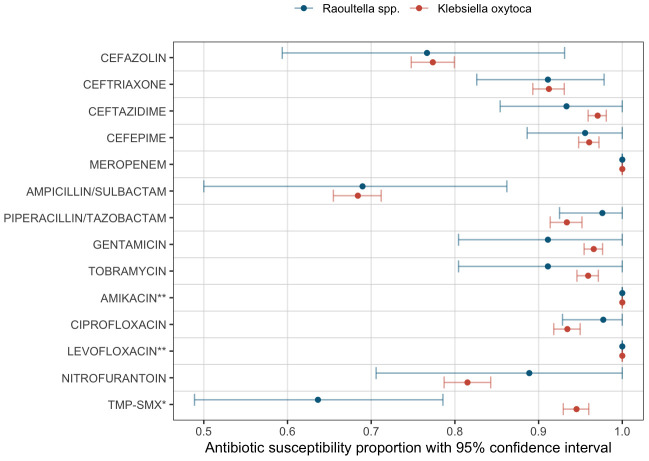
Antibiotic susceptibility. Proportions of susceptible specimens per each antibiotic tested are shown as dots (Blue: *Raoultella* spp., Red: *K. oxytoca*). The bars show the estimated 95% bootstrap confidence intervals for the proportion of susceptibility per each antibiotic agent. *: Trimethoprim/Sulfamethoxazole. **: Insufficient sample sizes (Amikacin: 4 samples in *Raoultella* spp. and 50 samples in *K. oxytoca*; Levofloxacin: 1 sample in *Raoultella* spp. and 4 samples in *K. oxytoca*.).

MIC50, MIC 90 and antibiotic susceptibility for different antibiotics are shown in **Table 3**. The number of specimens tested for each antibiotic is listed also shown in **Table 3**. Antibiotic susceptibility and 95% confidence intervals are also shown in **Figure 2**. All confidence intervals for antibiotic susceptibility overlapped between *Raoultella* spp. and *K. oxytoca* except for Trimethoprim/Sulfamexthoxazole. Both *Raoultella* spp. and *K. oxytoca* were very susceptible to meropenem, piperacillin/tazobactam, and ciprofloxacin. Although the sample sizes are small, both species were also susceptible to levofloxacin and amikacin. Both *Raoultella* spp. and *K. oxytoca* were less sensitive to cefazolin (MIC50 of 4 and 8, respectively and MIC90 of 64 for both) and ampicillin/sulbactam (MIC50 of 4 and 8, respectively and MIC90 of 32 for both).

**Table 3:** Antibiotic susceptibility.

Antibiotics	*Raoultella* spp.				*Klebsiella oxytoca*			
	Number of specimens	MIC50	MIC90	Proportion of susceptibility (95% CI)	Number of specimens	MIC50	MIC90	Proportion of susceptibility (95% CI)
CEFAZOLIN	28	4.00	64.00	0.77 (0.59, 0.93)	1428	8.00	64.000	0.77 (0.75, 0.8)
CEFTRIAXONE	43	1.00	1.00	0.91 (0.83, 0.98)	1432	1.00	1.000	0.91 (0.89, 0.93)
CEFTAZIDIME	43	1.00	5.20	0.93 (0.85, 1)	1432	1.00	1.000	0.97 (0.96, 0.98)
CEFEPIME	43	1.00	1.00	0.96 (0.89, 1)	1431	1.00	1.000	0.96 (0.95, 0.97)
MEROPENEM	43	0.25	0.25	1 (1, 1)	1432	0.25	0.250	1 (1, 1)
AMPICILLIN/ SULBACTAM	27	4.00	32.00	0.69 (0.5, 0.86)	1427	8.00	32.000	0.68 (0.65, 0.71)
PIPERACILLIN/ TAZOBACTAM	41	4.00	16.00	0.98 (0.92, 1)	1262	4.00	8.000	0.93 (0.91, 0.95)
GENTAMICIN	43	1.00	1.00	0.91 (0.8, 1)	1432	1.00	1.000	0.97 (0.95, 0.98)
TOBRAMYCIN	43	1.00	1.00	0.91 (0.8, 1)	1432	1.00	1.000	0.96 (0.95, 0.97)
AMIKACIN	4	2.00	2.00	1 (1, 1)	50	2.00	4.000	1 (1, 1)
CIPROFLOXACIN	42	0.25	0.25	0.98 (0.93, 1)	1431	0.25	0.250	0.93 (0.92, 0.95)
LEVOFLOXACIN	1	0.12	0.12	1 (1, 1)	4	0.25	0.775	1 (1, 1)
NITROFURANTOIN	17	16.00	41.60	0.89 (0.71, 1)	831	32.00	64.000	0.82 (0.79, 0.84)
TRIMETHOPRIM/SULFAMETHOXAZOLE	42	1.00	20.00	0.64 (0.49, 0.79)	1422	1.00	1.000	0.95 (0.93, 0.96)

## 4 Discussion

To the best of our knowledge, our study is the first study to compare the clinical differences between *Raoultella* spp. and *Klebsiella oxytoca* within the same study population. We found similarities in patient demographics (age and sex) and antibiotic susceptibility studies between the two bacteria species. However, there was a significant difference in disease severity measured by the proportion of ICU admissions within 48 hours of specimen collection. Patients with *Raoultella* positive cultures were significantly more likely to be admitted to ICU compared to patients with *Klebsiella oxytoca*. There were also significant differences in the culture sites. The frequency of urinary tract isolates was significantly higher among *Klebsiella oxytoca* patients than among *Raoultella* patients. At the same time, *Raoultella* patients were significantly more likely to have blood stream isolates, which likely represent blood stream infection. When only ICU admissions are compared, however, these differences were statistically insignificant.

An important microbiological difference between *Raoultella* spp. and *Klebsiella* spp. is known to be the ability of *Raoultella* spp. to produce histamines (Appel et al., 2021). Histamine is a potent proinflammatory molecule which plays a key role in initiating the immune response and is a possible contributor to cytokine storm and shock (Dale and Laidlaw, 1919; O’Mahony et al., 2011; Eldanasory et al., 2020). The higher proportion of ICU admissions among patients with *Raoultella* infection may be related to the histamine producing ability of *Raoultella* spp. Further studies are needed to investigate the role of *Raoultella*-produced histamine in patients’ disease severity.

Among different techniques to identify *Raoultella* species, MALDI-TOF is reported to be most reliable in distinguishing *Raoultella* from *Klebsiella* species (Ponce-Alonso et al., 2016). The application of MALDI-TOF in microbiological identification is relatively new (Singhal et al., 2015) and not every microbiology laboratory has this newer technology available. This may affect our analysis in two ways: 1) The difference between *Raoultella* spp. and *K. oxytoca* species may appear smaller, because some *Raoultella* specimens are misclassified as *K. oxytoca*. 2) If there were multiple microbiology laboratories using different detection techniques and certain specimens were more likely to be analyzed using MALDI-TOF, a bias arising from such systemic difference in detection techniques cannot be ruled out in our analysis.

There were more re-infection and repeat cultures among patients with *K. oxytoca* than patients with *Raoultella* (the number of specimens divided by the number of patients, 1.38 vs. 1.25). Because *Raoultella* is more readily identified using newer technologies, *Raoultella* positive cultures are likely to be more frequent towards the end of the study period. In this case, there was less time for patients with *Raoultella* infection to have a re-infection and repeat cultures. This may have contributed to the larger number of repeated cultures on average in *K. oxytoca* than in *Raoultella* spp.

Multidrug resistant *Raoultella* spp. is uncommonly reported (Mal et al., 2019; Tayo and Nyame, 2022). In our study, *Raoultella* spp. had high rates of susceptibility (>85%) to most broad-spectrum antibiotics, including carbapenem, third-generation cephalosporin, fluoroquinolones, aminoglycosides and trimethoprim/sulfamethoxazole. These observed rates were comparable to a case series of 187 isolates of *Raoultella ornithinolytica* (Seng et al., 2016). In addition, *Raoultella* spp. tended to have a higher rate of cefazolin susceptibility than *K. oxytoca* (72% vs. 52%), although statistical significance was not reached, likely due to the relatively small sample size of *Raoultella* spp. resulting in limited statistical power. Studies with a larger sample size are required to further determine the resistance profile of *Raoultella* species.

An important limitation of our study is selection bias. The patient population is likely skewed towards sicker patients as the inclusion criteria to the database we utilized are ED visits or ICU admissions. It is possible that sites of bacterial culture are strongly affected by the selection of patient population or the context of specimen collection (e.g. at ED or at ICU). Data from the outpatient setting is limited and patients with less severe infection are likely underrepresented in our study. Another limitation of our study is as mentioned above the unstandardized identification technique. Prior to the introduction of MALDI-TOF many *Raoultella* species are misclassified as *Klebsiella* species (Park et al., 2011). As a result, the differences between the two organisms may appear smaller and no difference found in our study does not prove that there are no differences between the two organisms. Furthermore, the relatively small number of patients with *Raoultella* positive cultures limits the statistical power of our analysis to detect differences between the two species. Finally, because we did not have access to the full clinical records (e.g., clinician’s notes) or to the microbiology samples, the presented results are limited by the data availability. Despite these limitations, our study adds clinically important information about the differences between the newly classified bacterial species, *Raoultella* spp., and its closest species according to the original classification, *K. oxytoca*, which has not been studied previously. Our study gives justification for future studies that are dedicated to compare these two species from clinical perspectives. Future studies should focus on isolates of *Raoutella* and *K. oxytoca* identified by standardized identification techniques such as MALDI-TOF or genome-sequencing and compare patients’ clinical courses prospectively. Developing a broader dataset of the clinical characteristics of *Raoultella* species in comparison to *Klebsiella* species will help us better understand this newly classified bacteria species and guide patient care.

## 5 Conclusion

While we did not find any significant differences in the patient demographics between *Raoultella* spp. and *K. oxytoca*, *Raoultella* species may cause more serious infection requiring ICU admissions. Also, *Raoultella* species may cause blood stream infection more frequently than *K. oxytoca.* Finally, *Raoultella* isolates were more often resistant to Trimethoprim/Sulfamethoxazole than *K. oxytoca*.

## Data availability statement

Publicly available datasets were analyzed in this study. This data can be found here: https://physionet.org/content/mimiciv/2.2/.

## Author contributions

SM: Conceptualization, Data curation, Formal analysis, Investigation, Methodology, Validation, Visualization, Writing – original draft, Writing – review & editing. NC: Conceptualization, Methodology, Writing – original draft, Writing – review & editing. RC: Conceptualization, Investigation, Methodology, Supervision, Writing – original draft, Writing – review & editing.
