# Supplementary material for: Clinical differences between Raoultella spp. and Klebsiella oxytoca
Source: Front Cell Infect Microbiol. 2024 Jun 3;14:1260212. doi: 10.3389/fcimb.2024.1260212 (PMC11180880; doi:10.3389/fcimb.2024.1260212)
Supplement: Supplementary file 1 [file Table_1.docx]

**Supplement**

**1. Missingness by antibiotics**

**a) Raoultella spp.**

| **Antibiotic** | **MIC value Missing** | **Number of observations** | **Proportion** |
| --- | --- | --- | --- |
| AMIKACIN | Non-missing observations | 4 | 1.000 |
| AMIKACIN | Missing observations | 0 | 0.000 |
| AMPICILLIN/SULBACTAM | Non-missing observations | 27 | 1.000 |
| AMPICILLIN/SULBACTAM | Missing observations | 0 | 0.000 |
| CEFAZOLIN | Non-missing observations | 28 | 1.000 |
| CEFAZOLIN | Missing observations | 0 | 0.000 |
| CEFEPIME | Non-missing observations | 41 | 0.953 |
| CEFEPIME | Missing observations | 2 | 0.047 |
| CEFTAZIDIME | Non-missing observations | 43 | 1.000 |
| CEFTAZIDIME | Missing observations | 0 | 0.000 |
| CEFTRIAXONE | Non-missing observations | 43 | 1.000 |
| CEFTRIAXONE | Missing observations | 0 | 0.000 |
| CIPROFLOXACIN | Non-missing observations | 42 | 1.000 |
| CIPROFLOXACIN | Missing observations | 0 | 0.000 |
| GENTAMICIN | Non-missing observations | 43 | 1.000 |
| GENTAMICIN | Missing observations | 0 | 0.000 |
| LEVOFLOXACIN | Non-missing observations | 1 | 1.000 |
| LEVOFLOXACIN | Missing observations | 0 | 0.000 |
| MEROPENEM | Non-missing observations | 43 | 1.000 |
| MEROPENEM | Missing observations | 0 | 0.000 |
| NITROFURANTOIN | Non-missing observations | 17 | 1.000 |
| NITROFURANTOIN | Missing observations | 0 | 0.000 |
| PIPERACILLIN/TAZO | Non-missing observations | 39 | 0.951 |
| PIPERACILLIN/TAZO | Missing observations | 2 | 0.049 |
| TOBRAMYCIN | Non-missing observations | 43 | 1.000 |
| TOBRAMYCIN | Missing observations | 0 | 0.000 |
| TRIMETHOPRIM/SULFA | Non-missing observations | 42 | 1.000 |
| TRIMETHOPRIM/SULFA | Missing observations | 0 | 0.000 |

**b) *Klebsiella oxytoca***

| **Antibiotic** | **MIC value Missing** | **Number of observations** | **Proportion** |
| --- | --- | --- | --- |
| AMIKACIN | Non-missing observations | 50 | 1.000 |
| AMIKACIN | Missing observations | 0 | 0.000 |
| AMPICILLIN/SULBACTAM | Non-missing observations | 1412 | 0.989 |
| AMPICILLIN/SULBACTAM | Missing observations | 15 | 0.011 |
| CEFAZOLIN | Non-missing observations | 1381 | 0.966 |
| CEFAZOLIN | Missing observations | 48 | 0.034 |
| CEFEPIME | Non-missing observations | 1382 | 0.966 |
| CEFEPIME | Missing observations | 49 | 0.034 |
| CEFTAZIDIME | Non-missing observations | 1423 | 0.994 |
| CEFTAZIDIME | Missing observations | 9 | 0.006 |
| CEFTRIAXONE | Non-missing observations | 1419 | 0.991 |
| CEFTRIAXONE | Missing observations | 13 | 0.009 |
| CIPROFLOXACIN | Non-missing observations | 1431 | 1.000 |
| CIPROFLOXACIN | Missing observations | 0 | 0.000 |
| GENTAMICIN | Non-missing observations | 1432 | 1.000 |
| GENTAMICIN | Missing observations | 0 | 0.000 |
| LEVOFLOXACIN | Non-missing observations | 4 | 1.000 |
| LEVOFLOXACIN | Missing observations | 0 | 0.000 |
| MEROPENEM | Non-missing observations | 1430 | 0.999 |
| MEROPENEM | Missing observations | 2 | 0.001 |
| NITROFURANTOIN | Non-missing observations | 831 | 1.000 |
| NITROFURANTOIN | Missing observations | 0 | 0.000 |
| PIPERACILLIN/TAZO | Non-missing observations | 1201 | 0.952 |
| PIPERACILLIN/TAZO | Missing observations | 61 | 0.048 |
| TOBRAMYCIN | Non-missing observations | 1432 | 1.000 |
| TOBRAMYCIN | Missing observations | 0 | 0.000 |
| TRIMETHOPRIM/SULFA | Non-missing observations | 1420 | 0.999 |
| TRIMETHOPRIM/SULFA | Missing observations | 2 | 0.001 |

**2. Missingness distribution by susceptibility**

**a) *Raoultella spp.***

| **Interpretation** | **MIC value Missing** | **Number of observations** | **Proportion** |
| --- | --- | --- | --- |
| I | Non-missing observations | 9 | 0.900 |
| I | Missing observations | 1 | 0.100 |
| R | Non-missing observations | 10 | 0.769 |
| R | Missing observations | 3 | 0.231 |
| S | Non-missing observations | 43 | 1.000 |

**b) *Klebsiella oxytoca***

| **Interpretation** | **MIC value Missing** | **Number of observations** | **Proportion** |
| --- | --- | --- | --- |
| I | Non-missing observations | 462 | 0.981 |
| I | Missing observations | 9 | 0.019 |
| R | Non-missing observations | 674 | 0.872 |
| R | Missing observations | 99 | 0.128 |
| S | Non-missing observations | 1432 | 0.962 |
| S | Missing observations | 56 | 0.038 |

**3. Blood stream infection**

**a) Summary of data and patient demographics**

|  | ***Raoultella spp.*** | ***Klebsiella oxytoca*** |
| --- | --- | --- |
| Number of patients | 10 | 134 |
| Number of specimen | 16 | 211 |
| Average age | 63.9 | 62.4 |
| Age quantiles | c(25% = 55, 50% = 58, 75% = 83) | c(25% = 53, 50% = 62, 75% = 74.5) |
| Number of female patients | F:5 | F:55 |
| Number of male patients | M:5 | M:79 |
| Number of patients whose specimen is collected during or within 48 hours prior to hospitalization | 10 | 130 |
| Number of patients whose specimen is collected during or within 48 hours prior to ICU admission | 6 | 51 |
| Number of specimens collected during or within 48 hours prior to hospitalization | 16 | 205 |
| Number of specimens collected during or within 48 hours prior to ICU admission | 10 | 80 |

**b) Antibiotic susceptibility**

| ***Raoultella spp.*** | | | ***Klebsiella oxytoca*** | | |  |
| --- | --- | --- | --- | --- | --- | --- |
| **Antibiotics** | **Number of specimens** | **MIC50** | **MIC90** | **Number of specimens** | **MIC50** | **MIC90** |
| CEFAZOLIN | 4 | 4.00 | 12.40 | 139 | 6.00 | 64.00 |
| CEFTRIAXONE | 9 | 1.00 | 1.00 | 140 | 1.00 | 2.00 |
| CEFTAZIDIME | 9 | 1.00 | 4.00 | 140 | 1.00 | 1.00 |
| CEFEPIME | 9 | 1.00 | 1.00 | 140 | 1.00 | 1.00 |
| MEROPENEM | 9 | 0.25 | 0.25 | 140 | 0.25 | 0.25 |
| AMPICILLIN/SULBACTAM | 4 | 3.00 | 23.60 | 139 | 8.00 | 32.00 |
| PIPERACILLIN/TAZO | 9 | 4.00 | 4.00 | 138 | 4.00 | 8.00 |
| GENTAMICIN | 9 | 1.00 | 4.00 | 140 | 1.00 | 1.00 |
| TOBRAMYCIN | 9 | 1.00 | 2.40 | 140 | 1.00 | 1.00 |
| AMIKACIN | 1 | 2.00 | 2.00 | 6 | 2.00 | 2.00 |
| CIPROFLOXACIN | 9 | 0.25 | 0.25 | 140 | 0.25 | 0.25 |
| TRIMETHOPRIM/SULFA | 9 | 1.00 | 16.00 | 137 | 1.00 | 1.00 |

**4. *Raoultella planticola* vs. *Raoultella ornithinolytica***

**a) Summary of data and patient demographics**

|  | ***Raoultella planticola*** | ***Raoultella ornithinolytica*** |
| --- | --- | --- |
| Number of patients | 30 | 13 |
| Number of specimen | 37 | 14 |
| Average age | 66.5 | 68.3 |
| Age quantiles | 25%: 58  50%: 67  75%: 77 | 25%: 52.5  50%: 70.5  75%: 82 |
| Number of female patients | F:16 | F:8 |
| Number of male patients | M:14 | M:5 |
| Number of patients whose specimen is collected during or within 48 hours prior to hospitalization | 26 | 11 |
| Number of patients whose specimen is collected during or within 48 hours prior to ICU admission | 12 | 7 |
| Number of specimens collected during or within 48 hours prior to hospitalization | 33 | 12 |
| Number of specimens collected during or within 48 hours prior to ICU admission | 16 | 8 |

**b) Culture sites**

|  | ***Raoultella ornithinolytica*** | | ***Raoultella planticola*** | |
| --- | --- | --- | --- | --- |
| **Culture site** | **Number of specimens** |  | **Number of specimens** | **Number of subjects** |
| Respiratory | 2 (25%) | 2 (28.6%) | 5 (35.7%) | 4 (33.3%) |
| Blood | 2 (25%) | 1 (14.3%) | 2 (14.3%) | 2 (16.7%) |
| Bile | 2 (25%) | 2 (28.6%) | NA | NA |
| Peritoneal fluid | 1 (12.5%) | 1 (14.3%) | 1 (7.1%) | 1 (8.3%) |
| Urine | 1 (12.5%) | 1 (14.3%) | 1 (7.1%) | 1 (8.3%) |
| Wound | NA | NA | 3 (21.4%) | 2 (16.7%) |
| Other | NA | NA | 2 (14.3%) | 2 (16.7%) |
